# Colonization with selected antibiotic resistant bacteria among a cohort of Sri Lankan university students

**DOI:** 10.1186/s12879-021-06289-z

**Published:** 2021-06-15

**Authors:** Thilini Munasinghe, Gihani Vidanapathirana, Shahlina Kuthubdeen, Asela Ekanayake, Sacheera Angulmaduwa, Kunchana De Silva, Susan Subhasinghe, Ruwani Kalupahana, Veranja Liyanapathirana, Margaret Ip

**Affiliations:** 1 Postgraduate Institute of Science, University of Peradeniya, Kandy, Sri Lanka; 2 Faculty of Medicine, University of Peradeniya, Kandy, Sri Lanka; 3 Department of Microbiology, Faculty of Medicine, University of Peradeniya, Kandy, Sri Lanka; 4 Department of Veterinary Public Health and Pharmacology, Faculty of Veterinary Medicine and Animal Science, University of Peradeniya, Kandy, Sri Lanka; 5 Department of Microbiology, Faculty of Medicine, The Chinese University of Hong Kong, Hong Kong, Hong Kong SAR

**Keywords:** MRSA, ESBL, CRE, Colonization, University students

## Abstract

**Background:**

Antibiotic Resistance is an imminent global public health threat. Antibiotic resistance emerged in healthcare settings and has now moved on to the community settings.

This study was conducted to identify the rates of asymptomatic colonization with selected antibiotic resistant organisms, (Methicillin Resistant *Staphylococcus aureus* (MRSA), Extended Spectrum Beta Lactamase (ESBL) producing *Escherichia coli* and *Klebsiella* spp and carbapenem resistant *E.coli* and *Klebsiella* spp) - among a group of university students in Sri Lanka. Identification of genetic determinants of MRSA and ESBL was an additional objective of the study.

**Methods:**

A self - collected nasal swab and a peri-rectal swab collected after passing stools were obtained. Routine microbiological methods were used for the isolation *S.aureus* from the nasal swab and *E.coli* and *Klebsiella* species from the peri-rectal swab. Antibiotic sensitivity testing was performed as recommended by clinical and laboratory standard institute (CLSI).

Three (3) genes that are responsible for ESBL production; *bla*_CTX-M_, *bla*_SHV_, and *bla*_TEM_ were tested using previously described primers and PCR procedures. Identification of *MecA* and *PVL* genes attributed to MRSA was also done with PCR.

**Results:**

A total of 322 participants between 21 and 28 years were recruited representing 5 different faculties of study. Seventy one (22.0%) were colonized with *S.aureus* and 14 among them with MRSA, making the MRSA colonization rate of 4.3%. Forty five (15%) of the participants were colonized with an ESBL producing *E.coli* or *Klebsiella* spp. No one was colonized with carbapenem resistant *E.coli* or *Klebsiella* species. Of the 45 ESBL producers the commonest genetic determinant identified was *bla*_CTX-M_ (*n* = 36), while 16 isolates had *bla*_TEM_ and 7 had *bla*_SHV*.*_ Similarly, of the 14 isolates identified as MRSA, 3 (21.4%) were found to be *PVL* positive while 11 (78.6%) were *MecA* positive.

**Conclusions:**

A high rate of colonization with ESBL producing *E.coli* and *Klebsiella* species was noted in our study group.

## Introduction

Antimicrobial resistance (AMR) has emerged as one of the principle public health concerns of this era. AMR has spread globally, compromising the ability to use antibiotics to treat infectious diseases. Resistant organisms, initially found in health care environments are now being increasingly found in community setting. Asymptomatic colonization with resistant organisms may subsequently cause serious infections in other sites of the given individual or in susceptible individuals [1].

AMR is a key challenge faced by the health care sector in Sri Lanka [2]. Along with the emergence of Gram negative resistance, resistance in Gram positive bacteria continue to pose a challenge. Infections caused by Methicillin Resistant *Staphylococcus aureus* (MRSA), ESBL producing enterobacteriaceae (ESBLE) and carbapenem resistant enterobacteriaceae (CRE) in hospital settings has been well studied in Sri Lanka [3]. ESBLE are emerging as causative agents in community onset infections [4]. However, data on colonization with these resistant bacteria among healthy young adults is limited. Colonized individuals may act as a reservoir for the spread of these bacteria in the community and studies have shown that people colonized with ESBLE are likely to contribute to others getting colonized with similar bacteria than non-human factors [5].

This study was conducted to identify the rates of colonization with selected antibiotic resistant organisms among a group of university students in Sri Lanka and to identify factors associated with such colonization.

## Materials and methods

This was a descriptive cross-sectional study which included undergraduate students of University of Peradeniya between 18 to 28 years of age. Nasal swabs and peri-anal swabs were collected from the participants from December, 2017 to October 2018. Ethical approval for the study was obtained from the Ethics Review Committee, Faculty of Medicine, University of Peradeniya (2017/EC/29).

### Subject enrolment and sample collection process

Three hundred twenty-two participants were recruited using convenience sampling method. Samples were self-collected by the participants. They were provided with two swabs. Participants were advised to insert one swab in to both nostrils, rotate to touch all sides of the nasal wall and insert back to the sheath. For collecting the peri-anal swab, participants were advised to insert the swab up to 1 cm through the anal orifice and touch the anal wall, followed by touching the peri-rectal area, after passing stool, before cleaning. The collected specimens were transported to the laboratory within 2 hours after collection.

### Laboratory procedure

Collected nasal swabs were processed to isolate *Staphylococcus aureus* using conventional laboratory techniques including 6.5% NaCl tolerance, growth on Manitol Salt Agar plates, Gram staining, catalase test, slide and tube coagulase tests and DNase test.

Peri-anal swabs were processed to isolate ESBL producing Enterobacteriaceae species. The identification of the *Escherichia coli*, *Klebsiella pneumoniae* or *Klebsiella oxytoca* species were based on the Cowan and Steel’s manual [6]. Quality-controlled MacConkey agar plates supplemented with ceftazidime (1 mg/L) were used for the tentative identification of ESBL producers.

Organisms thus identified were inoculated on 15–20% glycerol broth and stored in − 80 °C till further testing.

### Antibiotic sensitivity testing (ABST)

ABSTs were done using disc diffusion method following the Clinical and Laboratory Standard Institute (CLSI) guide [7]. For *Staphylococcus aureus,* the antibiotics used were cefoxitin (30 μg), gentamicin (10 μg), ciprofloxacin (5 μg), clindamycin (2 μg), fusidic acid (5 μg) and erythromycin (15 μg). For *E.coli* and *Klebsiella* species, the antibiotics used for the study were cefotaxime (30 μg), ceftazidime (30 μg), imipenem (10 μg), meropenem (10 μg), aztreonam (30 μg), gentamicin (10 μg), ciprofloxacin (5 μg) and levofloxacin (5 μg).

Isolates which were with zone diameters ≤21 mm for cefoxitin (30 μg) for *Staphylococcus aureus* were considered as MRSA isolates [7].

Isolates with zone diameters ≤22 mm and ≤ 27 mm for antibiotics ceftazidime (30 μg) and/or cefotaxime (30 μg) respectively for both *Escherichia coli.* and *Klebsiella* spp. were considered as fulfilling ESBL screening criteria for ESBL production. ESBL confirmatory testing was performed on these isolates. Ceftazidime (30 μg) and cefotaxime (30 μg) were used alone and in combination with clavulanic acid [ceftazidime (30 μg)/ ceftazidime-clavulanate (30/10 μg) and cefotaxime (30 μg/ cefotaxime-clavulanate (30/10 μg)] were used for the confimatory testing. The results were interpreted according to CLSI guidelines [7].

### DNA extraction

Simple boil lysis method was used to extract the DNA from the identified MRSA and ESBL species.

### Identification of genetic determinants of ESBL production

Three (3) genes that are responsible for ESBL production; *bla*_CTX-M_, *bla*_SHV_, and *bla*_TEM_ were tested using previously described primers and PCR procedures [8].

### Identification of PVL and mecA genes

Previously described primers were used for identification of *MecA* (162 bp) and *PVL* (85 bp) genes respectively [9].

### Data analysis

SPSS software version 22 was used for data analysis (IBM statistics). A *p* value of < 0.05 was taken as being statistically significant. Percentage of MRSA and ESBL colonization among the study population was calculated. Factors associated with antibiotic resistant bacteria were identified using Chi square test or the Fisher’s Exact test. Of the ESBL producers, proportions of isolates carrying each genetic determinant, and proportion of MRSA isolates with *PVL* gene and *MecA* genes were calculated.

## Results

### Demographic details

During the study period, a total of 322 samples were collected. The participants’ ages ranged from 20 to 28 years (Mean: 23.7 years, SD 1.4). The numbers of female and male students were 166 (51.6%) and 156 (48.4%) respectively. Of the 322 participants, 210 (65.2%) were from health science related faculties and 112 (34.8%) were from non-health related faculties. The number of participants residing at university residential facilities were 225 (69.9%) and 97 were (30.1%) day scholars. There were 136 (42.2%) participants who used antibiotics during the 3 months preceding sample collection and 15 (4.7%) participants were having chronic diseases.

### Colonization with *Staphylococcus aureus* and MRSA

Among the 322 participants, *Staphylococcus aureus* was isolated from 71 (22%) participants. This included 14 (4.3%) participants who were colonized with Methicillin Resistant *Staphylococcus aureus* (MRSA) isolates.

Nine of the 156 males (5.8%) and 5 of the 166 females (3%) were colonized with MRSA. None of the factors studied were significantly associated with colonization with MRSA (Table 1).

**Table 1:** Association of variables with MRSA

Variables		Number of participants (*n* = 322)		Significance*
		Participants colonized with MRSA	Participants not colonized MRSA	
Sex	Male (*n* = 156)	9 (5.8%)	147 (94.2%)	0.28*
	Female (*n* = 166)	5 (3.0%)	161 (97.0%)	
Faculty	Health Science related (*n* = 210)	10 (4.8%)	200 (95.2%)	0.78^
	None Health related (*n* = 112)	4 (3.6%)	108 (96.4%)	
Antibiotic intake	Yes (*n* = 133)	8 (6.0%)	125 (94.0%)	0.27*
	No (*n* = 189)	6 (3.2%)	183 (96.8%)	
Chronic diseases	Yes (*n* = 15)	1 (6.7%)	14 (93.3%)	0.49^
	No (*n* = 307)	13 (4.2%)	294 (95.8%)	
Residence	Residents (*n* = 225)	7 (3.1%)	218 (96.9%)	0.09^
	Day scholars (*n* = 97)	7 (7.2%)	90 (92.8%)	

*p value calculated using the Chi-square test

^*p* value calculated using Fisher’s Exact test

### Antibiotic sensitivity testing of *Staphylococcus aureus* and MRSA isolates

Of the 71 participants colonized with *Staphylococcus aureus*, 57 were colonized with Methicillin Sensitive *Staphylococcus aureus* (MSSA), while 14 were colonized with MRSA. Their sensitivities are given in Table 2.

**Table 2:** Susceptibility rates for MRSA and MSSA strains

	MSSA strains (*n* = 57)	MRSA strains (*n* = 14)
Gentamicin (10 μg)	54 (94.7%)	13 (92.9%)
Ciprofloxacin (5 μg)	42 (73.7%)	10 (71.4%)
Clindamycin (2 μg) Including inducible resistant isolates	50 (87.7%)	11 (78.6%)
Clindamycin (2 μg) Excluding inducible resistant isolates	50 (87.7%)	14 (100.0%)
Fusidic acid (5 μg)	49 (86.0%)	12 (85.7%)
Erythromycin (15 μg)	24 (42.1%)	4 (28.6%)

### Identification of PVL and MecA genes

Of the 14 isolates identified as MRSA, 3 isolates were found to be *PVL* positive while 11 were *MecA* positive.

### Isolation of *Escherichia coli* and *Klebsiella* species from ESBL screening plates

Number of samples which yielded Gram negative bacilli was 108 (33.5%). Of these 108 cultures 134 Gram negative bacterial isolates were obtained. There were 65 (48.5%) *E. coli* isolates and 3 (2.2%) *Klebsiella pneumoniae* isolates while others were Gram negative cocci and were not included in further analysis.

### Colonization rates for ESBL producers

Forty five of the 322 (14.0%) participants were colonized with ESBL producing *E. coli* (44, 13.7%) or *K. pneumoniae* (1, 0.3%) isolates.

### Factors associated with colonization with ESBL producers

Twenty four of the 156 males (15.4%) and 21 of the 166 females (12.7%) were colonized with ESBL producers. This difference was not statistically significant (*p* = 0.52, Chi square test). Having a chronic disease was significantly associated with colonizing with ESBL producers (33.3% colonization among those with chronic diseases vs 13.0% among those without chronic diseases) (Table 3).

**Table 3:** Association of variables with colonization by ESBL producers

Variables		Number of participants (*n* = 322)		Significance*
		Participants colonized with ESBL	Participants not colonized ESBL	
Sex	Male (*n* = 156)	24 (15.4%)	132 (84.6%)	0.52*
	Female (*n* = 166)	21 (12.7%)	145 (87.3%)	
Faculty	Health Science related (*n* = 210)	31 (14.8%)	179 (85.2%)	0.35*
	None Health related (*n* = 112)	14 (12.5%)	98 (87.5%)	
Antibiotic intake	Yes (*n* = 136)	23 (17.3%)	110 (82.7%)	0.19*
	No (*n* = 186)	22 (11.6%)	167 (88.4%)	
Chronic diseases	Yes (*n* = 15)	5 (33.3%)	10 (66.7%)	0.04^
	No (*n* = 307)	40 (13.0%)	267 (87.0%)	
Residence	Residents (*n* = 225)	30 (13.3%)	195 (86.7%)	0.72*
	Non-residents (*n* = 97)	15 (15.5%)	82 (84.5%)	

*p value calculated using the Chi-square test

^*p* value calculated using Fisher’s Exact test

### Antibiotic sensitivity testing

All ESBL producing isolates were susceptible for carbapenems. Antibiotic sensitivity rates for ESBL producers are given in Table 4.

**Table 4:** Antibiotic susceptibility rates of ESBL producers

	ESBL producers N (%)
Cefotaxime (30 μg)	0 (0.0%)
Ceftazidime(30 μg)	1 (2.2%)
Aztreonam (30 μg)	1 (2.2%)
Meropenem (10 μg)	45 (100%)
Imipenem (10 μg)	45 (100%)
Ciprofloxacin (5 μg)	18 (40.0%)
Levofloxacin (5 μg)	22 (48.8%)
Gentamicin (10 μg)	43 (95.5%)

### Genetic determinants of ESBL producers

Of the 45 ESBL producers the commonest genetic determinant identified was *bla*_CTX-M_ (*n* = 36), while 16 isolates had *bla*_TEM_ and 7 had *bla*_SHV*.*_ (Table 5).

**Table 5:** Genetic determinants found on ESBL producers

Gene	No of isolates
*bla*_*SHV*_ only	0
*bla*_*TEM*_ only	3
*bla*_*CTX-M*_ o*nly*	29
*bla*_*SHV+*_ *bla*_*TEM*_	6
*bla*_*CTX-M+*_ *bla*_*SHV*_	0
*bla*_*CTX-M+*_ *bla*_*TEM*_	6
*bla*_*CTX-M+*_ *bla*_*TEM+*_ *bla*_*SHV*_	1
Total	45

### Colonization rates for CRE

All isolates were sensitive to imipenem and meropenem. None of the 322 (0.0%) participants were colonized with CRE.

### Co-colonization with MRSA and ESBLE

Only one (0.31%) participant was co-colonized with both MRSA in nasal region and ESBLE in the peri anal region.

## Discussion

In the current study, we have assessed colonization of Methicillin Resistant *Staphylococcus aureus* (MRSA), extended spectrum beta lactamase producing enterobacteriaceae (ESBL) and carpabenem resistant enterobacteriaceae (CRE) among a selected group of university students in Sri Lanka.

According to the present study, rate of colonization of MRSA was 4.3%. Of the 14 isolates identified as MRSA, 3 (21.4%) were found to be *PVL* positive while 11 (78.6%) were *MecA* positive.

In this study MRSA colonization was higher in health science related students (4.8%) than in non-health science related students (3.6%). However, this difference was not statistically significant.

A similar study has been carried out in Rajarata University of Sri Lanka, located in another province of Sri Lanka, in order to evaluate the relationship between the exposure to healthcare settings and colonization with methicillin-resistant *Staphylococcus aureus* among medical students. The percentage of MRSA colonization found before clinical exposure and after 2.5 years of exposure was 6.36 and 49.57%, respectively [10]. However, the MRSA colonization rate after the clinical exposure was relatively higher than to the present study. Our study sample consisted of students from both health and non-health related faculties, and we did no check the exposure to clinical settings in health-related students. Therefore, we cannot compare the effect of health care facility exposure. However, the overall colonization rate in our study, and the colonization rate among non-clinical students in the cited study are similar.

There are a number of similar studies conducted in other countries with regards to the MRSA colonization among university students. A study detected the prevalence of MSSA and MRSA among medical students in Saudi Arabia, using molecular approaches revealed a colonization rate of 18.7% for *Staphylococcus aureus* and 6.7% for MRSA [11]. These rates are similar to what we found through conventional culture. Prevalence of nasal carriers of MRSA has been evaluated in a group of medical students in Jordan and 2.4% MRSA colonization was found [12]. According to the findings of the study, *S. aureus* nasal colonization was significantly associated with male gender and chronic illnesses. All the MRSA isolates were positive for *mecA* gene. Higher rates of *mecA* positive MRSA was observed in our study as well. Nevertheless, there were no significant factors associated with MRSA colonization in our study. Another study done in Taiwan on prevalence and the risk factors for MRSA colonization among adults in community settings has given a colonization rate of 3.8% [13]. It is stated that *S. aureus* has a specific niche preference for the anterior nostrils in adults [14]. This nasal carriage rate was also reported approximately 10% in the community [15]. Present study also supported the fact that nasal carriage has become a mode of persistence and may contribute to the spread of MRSA.

The reported prevalence of colonization with MRSA differs between institutions and geographic regions which might be due to differences in terms of study design, sample size and methods, samples used for MRSA detection. In addition to that, differences in actual colonization or changes in detection due to sample collection (nasal vs nasal, axillary, throat and groin) and processing methods might have affected the deviation in the colonizing rates.

When considering the antibiotic susceptibility rates of the isolated *Staphylococcus aureus*, the highest resistance was observed for erythromycin in both MSSA and MRSA. This was observed in another study where all the MRSA isolates were resistant to penicillin and erythromycin [16]. In another study done among school children in Ethiopia, susceptibility rates of gentamicin and erythromycin were 84.6 and 38.5% for MRSA and 98.5 and 82.3% for MSSA respectively [17]. These correlate with our study findings. MRSA globally has become multi-drug resistant due to acquisition of other genetic determinants of resistance [18].

The sensitivity of CA-MRSA related to infections was also found to be quite similar to findings of the current study. According to a study done relate to skin and soft tissue infections, MRSA susceptibility to erythromycin and clindamycin were 23.1 and 80.7% respectively [19]. Susceptibility rates in the study done in tertiary care hospital for erythromycin, clindamycin, ciprofloxacin, gentamicin was 35, 48.8, 28 and 32% [20].

Unlike community acquired MRSA (CA-MRSA), healthcare associated MRSA (HA-MRSA) has been studied extensively in Sri Lanka. Analysis of *Staphylococcus aureus* isolates obtained from a private hospital has identified an MRSA proportion of 42% [21]. Majority of the isolated MRSA had antibiograms compatible with community acquired MRSA which indicates the possibility of crossing over of antimicrobial resistance from health care settings into the community in Sri Lanka. In another study conducted to compare molecular characteristics of CAMRSA and HAMRSA strains isolated at the National Hospital of Sri Lanka, the proportion of HA-MRSA was 79% while CA-MRSA was 21%. The proportion of PVL gene among HA-MRSA isolates was 3.8% whereas proportion of *PVL* among CA-MRSA isolates was 95.2% [22]. In our study, the proportion of PVL positive isolates was more similar to the proportion reported from HA-MRSA isolates in this study.

However, it is now evident that there are lineages of CA-MRSA that are *PVL* negative. These clones have also shown a global distribution [23]. As we did not do in-dept molecular analysis on our isolates, we are unable to comment on this. However, the low rate of PVL prevalence does not exclude the isolates from being community origin.

In this study, the rate of colonization of ESBL producers was (*n* = 45) 14.0% and all except one isolate were *E. coli*. Of the 45 ESBL producers the commonest genetic determinant identified was *bla*_CTX-M_ (*n* = 36), while 16 isolates had *bla*_TEM_ and 7 had *bla*_SHV*.*_

Our ESBL colonizing rates were parallel to several other studies found in literature. In a study done among Mozambican university students, the proportion of students colonized with community acquired ESBL producers was found to be 20% [24]. Another study done among children attending pre-school childcare facilities in Laos has identified 23.2% of the study population to be colonised with ESBL producers [25]. The rate of colonization we found in the current study is much higher than the rate of vaginal colonization found among pregnant females presenting for delivery at Teaching Hospital Peradeniya [26]. The differences in sampling sites and study cohorts may be possible reasons for this difference. Further, a preliminary study in Switzerland has shown that vagina-perineal swab has a lower sensitivity of detecting ESBL carriage in pregnant women. Further, the ESBL carriage among pregnant women has found to be lower than the general population. Both these also could explain the lower rates of the study by Nanayakkara et al. compared to the current study [27].

In the present study, none of factors studied were significantly associated with colonization with ESBL producers other than presence of chronic disorders. Prevalence of ESBL producers was significantly high (33.3%) in students who suffer from chronic diseases than in students who did not (13%). Co-morbid conditions are a recognized risk factor for colonization with ESBL producers [28]. Frequent exposure of individuals with chronic diseases to health care settings might be the reason behind this. Previous exposure to antibiotics increases the chance of getting colonized with resistant bacteria. While the difference between those who recalled having used antibiotics in the 3 months before the study and those who did not, was not statistically significant, the former group had a higher colonization rate in the current study.

The susceptibility rates of ESBL producers for cefotaxime, ceftazidime, aztreonam, meropenem, imipenem, ciprofloxacin, levofloxacin and gentamicin were 0.0, 2.2, 2.2, 100, 100, 40, 48.8, and 95.5%. The study done among Mozambican university students, *E. coli* and *Klebsiella* spp. combined demonstrated 63 and 34% resistance rates of ceftazidime and ciprofloxacin respectively [24].

All ESBL producers were not resistant to both cefotaxime and ceftazidime which emphasizes the need to use both drugs in screening for ESBL production. The lower rate of susceptibility to levofloxacin is a reason to be concerned.

Among the ESBL producing enterobacteriaceae, the commonest genetic determinant of resistance currently is *bla*_CTX-M_ which is in agreement with our findings.

According to the current study there was no colonization of CRE among the study population. This is supported by several studies done on community acquired CRE. Study on colonization with ESBLE and CRE in international travellers returning to Germany has detected no CRE [29]. Another study done in children attending pre-school child care facilities in Laos also observed no CRE [25]. Kumudunie et al. has reported an alarming rate of CRE and the emergence of *bla*_KPC_ harboring *K. pneumoniae* in Sri Lanka among clinical isolates [30]. Other clinical studies have also demonstrated higher number of CRE [31]. This emphasizes the need of a further studies and continuous monitoring of CRE in the community.

We did not sequence the potential genetic determinants of ESBL production. This maybe a limitation as some narrow spectrum beta lactamases may also be identified through these primers. Further our samples were self-collected. Then though this may introduce a heterogeneity, self-collected nasal swabs and peri-rectal swabs have been shown to be effective methods in community surveillance [32–34]((Gambin 2012, Hogan 2016, Sun 2017). Further, peri-rectal swabs have also been shown as an effective alternative to collecting stool samples and used in multiple studies previously [35, 36] (Lautenback 2005, Blythe 2016).

## Conclusion

The asymptomatic colonization rate of CA-MRSA was 4.3% in the studied group of university undergraduates while the asymptomatic colonization rate of ESBL producers was 14% including 13.7 and 0.3% of *E. coli* and *K. pneumoniae* respectively. CRE was not found as a colonizer among the study group.

## Data Availability

The datasets used and analysed during the present study are available on the corresponding author on reasonable request.

## Abbreviations

MRSA: Methicillin resistant *Staphylococcus aureus*
MSSA: Methicillin Sensitive *Staphylococcus aureus*
CA-MRSA: Community Acquired Methicillin resistant *Staphylococcus aureus*
HA-MRSA: Healthcare Associated Methicillin resistant *Staphylococcus aureus*
ESBL: Extended Spectrum Beta Lactamase
ESBLE: ESBL producing enterobacteriaceae
CRE: Carbapenem Resistant Enterobacteriaceae
CLSI: Clinical and Laboratory Standard Institute
PCR: Polymerase Chain Reaction
AMR: Antimicrobial resistance
DNA: Deoxyribonucleic acid
